# Common sense: folk wisdom that ethnobiological and ethnomedical research cannot afford to ignore

**DOI:** 10.1186/1746-4269-9-80

**Published:** 2013-12-02

**Authors:** Thomas C Erren, Melissa S Koch, V Benno Meyer-Rochow

**Affiliations:** 1 Environmental Medicine and Prevention Research, Institute and Policlinic for Occupational Medicine, University Hospital of Cologne, Kerpener Str. 62, D-50937, Cologne, Germany; 2 Faculty of Science and Technology, Jacobs University Bremen, Campus Ring 6, D-28797, Bremen, Germany; 3 Department of Biology, Oulu University, SF-90300, Oulu, Finland

## Abstract

Common sense [CS], especially that of the non-scientist, can have predictive power to identify promising research avenues, as humans anywhere on Earth have always looked for causal links to understand, shape and control the world around them. CS is based on the experience of many individuals and is thus believed to hold some truths. Outcomes predicted by CS are compatible with observations made by whole populations and have survived tests conducted by a plethora of non-scientists. To explore our claim, we provide 4 examples of empirical insights (relevant to probably all ethnic groups on Earth) into causal phenomena predicted by CS: (i) “humans must have a sense of time”, (ii) “at extreme latitudes, more people have the winter blues”, (iii) “sleep is a cure for many ills” and (iv) “social networks affect health and disease”. While CS is fallible, it should not be ignored by science – however improbable or self-evident the causal relationships predicted by CS may appear to be.

## Background

### Common sense: what it is and how it can be defined

Numerous books and reviews have been devoted to the topic of common sense, henceforth abbreviated CS [1-4]. Unsurprisingly, ethnobiological and ethnomedical researchers, who gather information from human respondents, are regularly confronted with CS versus scientific proof when investigating people’s views and behaviour. For Australian Aborigines when bitten by a poisonous snake, it was CS to “sleep away the poison” and to rest for several days with the bitten limb covered with sand or earth [5]. Diseases and epidemics, according to CS of the Trobriand Islanders, arrive with gusts from the east [6] and members of the Ao-Naga in North-East-India told one of the authors (VBM-R) that all one needed to understand why insects could see at night was “because they cannot close their eyes”. For the Orang Asli of Malaysia no scientific explanation is needed why children and women must only consume small animals while men may also eat the larger species: CS tells them that the spirits of the larger animals can be inactivated by the stronger men, but not the weaker children and women [7]. In China traditionally women will not wash their hair for a month and avoid certain foods after having given birth to a baby [8] and in many parts of the world it is common sense to link the dawn of a new day with the cock’s crow [9]. CS finds itself expressed in idioms and proverbs, which, however, are frequently contradictory (e.g., “Birds of a feather flock together”, but “Opposites attract each other” or “You don’t teach an old dog new tricks”, but “You are never too old to learn”) and show the difficulty of dealing with CS. In this context CS bears similarities to the common law, for which Hoebel [10] in his book on “The Law of Primitive Man”, cites Holmes’ classic dictum “The life of law has not been logic; it has been experience”.

If CS were not an important asset (for scientists and non-scientists alike) it should not exist. From every part of the world, in every society, examples of CS abound, but what exactly *is* CS? Can it be defined? It may not come as a surprise that Nobel Prize laureates such as Albert Einstein and George Bernard Shaw have emphasized the value of CS. According to Einstein, CS is in and by itself a discipline, which should not be taken for granted. He asserts,

*“Common sense invents and constructs no less in its own field than science does in its domain.”*[11].

Shaw goes beyond that and suggests that although CS may be on the surface intuitive, it also is the foundation of unparalleled intellect, by stating

*“Commons sense is instinct. Enough of it is genius.”*[12].

Politically, *Common Sense* by Thomas Paine [13] is considered the most influential tract of the American Revolution. This 48-page pamphlet, with the enormous circulation of some 500,000 copies sold during 1776 alone, provided the American colonists with a powerful argument for independence from Britain. Paine understood that what populations think and believe can be a powerful resource and incentive. But, as pointed out earlier, to define CS is no easy task and even if we stated that CS refers to a generally accepted set of beliefs with regard to some topic or theme held by most people, the definition is not all-encompassing.

Historically, Aristotle described CS (Latin: *sensus communis*) as a person’s ability or power to judge or integrate the various impressions this person has gathered through his or her external senses in order to derive commonalities in them. In the 18^th^ century, the Scottish School of Common Sense argued that CS-beliefs determine our lives and are built upon a common understanding. Today’s purist’s meaning of the constructed term yields what people in common would agree on “makes sense”, produces a “gut feeling”. In this vein, CS is equivalent to what people believe or are convinced they know. And yet, in the end we have to say with Olson [14] “Common sense is like sanity; everybody needs it, but nobody can define it”.

In the following we shall examine if “everybody” (and in particular the ethnobiological and ethnomedical scientist interested in community health) really needs CS by focusing on CS in health-related issues. We feel that this gives us an opportunity to, at least, for our chosen field of inquiry to examine if CS is of any value to the scientist and therefore must not be dismissed when encountered in ethnobiological or ethnomedical inquiries, whether these take place in faraway and remote places or in our own countries and societies.

In principle, non-scientists and scientists alike develop CS, but scientists learn to question, to probe, to examine, and to discard what cannot be proven. The question therefore is “Can a non-scientist’s CS be a source and guide for innovative science?”

### The common sense of scientists and non-scientists

Scientists build CS on detailed knowledge – in theory and practice – of how to approach certain problems. They do well with problem-solving, because they know general principles on which to base solutions and how to apply these solutions to variations of known and already researched problems. All in all, scientists are experts in certain areas and, in principle, the term “expert” sounds positive and good to many. However, when it comes to identifying promising research avenues, which may yield causal insights for science, the expert status of scientists and their specific CS will not necessarily prove to be better than that of the non-expert’s or non-scientist’s CS [15]. Indeed, a scientist’s CS may serve him/her ill for two main reasons.

First, the scientist’s own interests and natural intuition could lead him or her to a specific and often narrow field of study. It is within this field that his/her CS develops. To now identify promising research avenues beyond one’s own could jeopardize the scientist’s status, career, and way of life. His/her work may appear misguided, opportunities to present findings at meetings or in journals may diminish, and funds to support research in an outdated field may dry up. The scientist’s specific CS will therefore tell him/her that it is in his/her vested interest not to identify promising research avenues beyond his/her own field(s).

Even worse than failing to identify promising research avenues is to actively block them [16]. To achieve the latter, scientists can prejudge perspectives as bleak – say that a novel line of investigation will lead nowhere, thus preventing innovative research from being pursued. Ethnobiology and ethnomedicine had to struggle with this kind of attitude as fledgling disciplines, trying to assert themselves against older established fields.

Second, if a scientist’s CS were to exclusively guide him/her in his/her research, this would be a recipe for delaying scientific progress. Indeed, if science were to proceed merely along what the CS of its professional protagonists suggests, we would likely look at infinitesimally small advances. Major leaps in knowledge were made when unexpected paths and improbable directions were taken rather than what a scientist’s professional consensus would have recommended. As molecular biologist and Nobel Prize laureate Max Perutz [17] asserts, “Discoveries cannot be planned; they pop up, like Puck, in unexpected corners”. And Horrobin [15,16] argues:

“Most scientists seem to be under the impression that the best hypotheses are those which seem most likely to be true. I follow Karl Popper in seeing the virtues of improbability. If a hypothesis which most [scientists or experts] think is probably true does turn out to be true (or rather is not falsified by crucial and valid experimental tests) then little progress has been made. If a hypothesis which most think is improbable turns out to be true then a scientific revolution occurs and progress is dramatic.”

**Figure 1:**
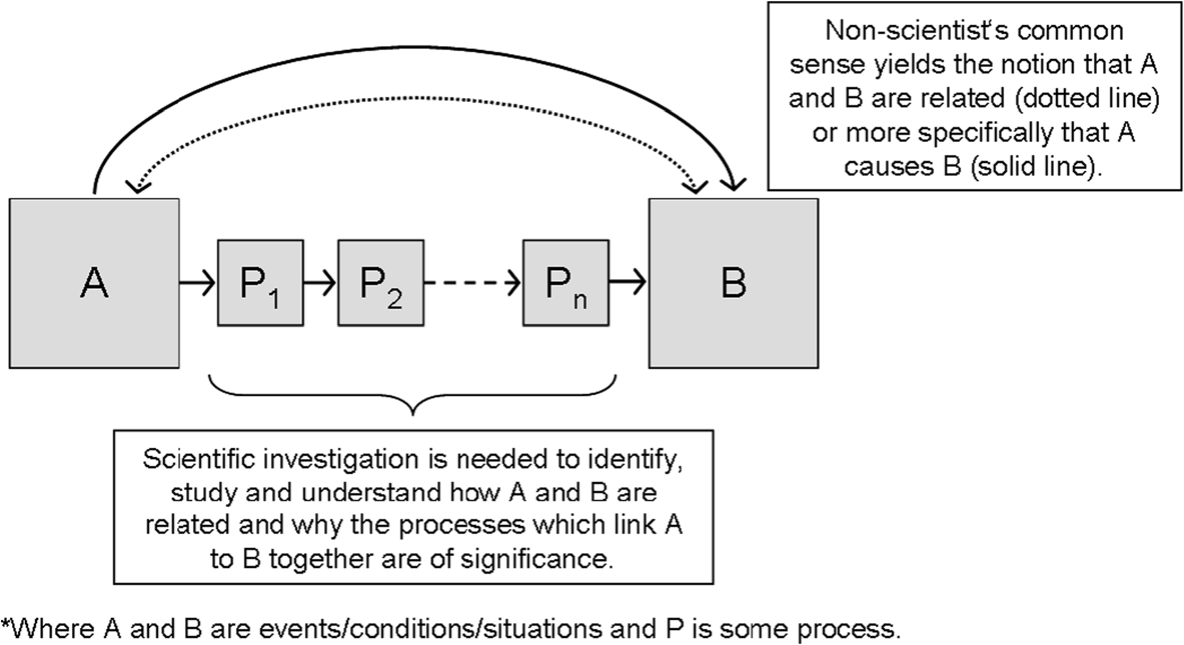
Where A and B are events/conditions/situations and P is process.

Although on the surface the “relationships” presented by the non-scientists’ CS may seem rather straightforward, deeper scientific investigation may reveal the counter-intuitive processes Horrobin [15] refers to (see Figure 1).

The non-scientist’s CS differs considerably from that of the scientist. For one, the CS of non-scientists, and how it is used and applied, is not infused with vested work and career interests, but rather has been developed, shaped and put under scrutiny by a large population. Indeed, much of the CS of populations is the result of powerful tests by many individuals of hypotheses in both time and space. In this vein, the non-scientists’ CS is the result of evolutionary steps or refinements along the way by the masses and across generations. Therefore, the CS of populations ultimately benefits from what has been coined “group intelligence” and “group creativity” [18].

There are, of course, interfaces between a non-scientist’s and a scientist’s CS. After all, scientists are, as a tiny (but at times powerful) minority, part of the CS evolutionary process within populations. In principle, they can persuade their immediate environment and, ultimately, whole populations if there are CS errors which need to be corrected. To achieve this end, scientists must want to share, write and speak about “scientific” truths so that non-experts understand them [19]. In reality, it can be a risky and dangerous undertaking to confront CS, especially in isolated, traditional communities and the provocative question has been asked “which behaviours do people explain?” [20].

Horrobin [21] declares, “While specialization in research is essential, obscurity in the presentation of specialized ideas is not.” Thus while research needs to be concise and very specific, communicating the findings requires straight forward terms that are formulated in a manner easily understood by the masses. This facilitates future research support. However, insights as to what frequently deters scientists from doing this can be prompted by Dennett, “There is a certain cachet in being hard to understand and being inaccessible. This is the way you make your reputation, by being obscure” [22]. In conjunction with this claim, Hillis goes into greater detail regarding the phenomenon:

*“It’s also true that 'popularizer’ is a pejorative term among scientists generally. A popularizer is somebody who explains what the issues are in ways people can understand. I think that it’s ridiculous that scientists don’t respect such people. In any other field, explaining to a congressional committee why what you’re doing is exciting and wonderful would be considered a service to the field. In science, you’re treated almost like somebody who has betrayed the secret club”*[22].

Of course, to what extent Dennett’s and Hillis’ observations of the scientific community hold true, varies among individual scientists and research groups. However, that scientists are often hesitant to broadly present their findings or share their views can be considered a disservice to the common good.

### On the theoretical value of the non-scientist’s common sense for science

We propose that the non-scientist’s CS can be a good resource for identifying causal chains between some A and B, which are – with some probability – worthy of scientific investigation. It follows that scientists should take note of the non-scientist’s CS to possibly identify promising research avenues. Granted, CS will regularly be confined to the observation of one (or more) final factor(s) acting in a causal mechanism. Notwithstanding, if researchers were to rigorously investigate *how* A leads to B, this could provide us with incentives and possibilities to manipulate chains of causation, which are of interest to many people. After all, the causal phenomena have already made it into the public’s awareness so that understanding how to shape and control links in such chains of causation should be welcomed. Had Galileo heeded what the fishermen’s CS said all along, he would not have failed to propose a correct theory of the tides [23].

In the following, we provide four empirical examples to explore how, through meticulous work, core science has uncovered causal details of what was – according to CS – linked all along:

(i) “There must be a sense of time in humans”;

(ii) “At the extremes of latitude, more people have the winter blues”;

(iii) “Sleep is a cure for many ills”;

(iv) “Social networks affect health and disease in man”.

### On the empirical value of the non-scientist’s common sense for science

(i) Research into the “sense of time” in humans

Even the earliest tribes understood that all living things, including humans, follow a rhythm; implying that there had to be some time-keeping system or clock driving rhythmic actions or functions to occur at regular intervals and according to the eloquent reasoning of Scharf [24] it is this sense of time in combination with memory and anticipation that led to verbal communication. Hunters and gatherers were in tune with the daily and seasonal cycles of the animals and plants they sought; had they not been, it would have made it quite difficult for human societies to function and grow. They were aware of tidal rhythms, lunar cycles and menstrual periodicities and often possessed food taboos in connection with timely recurring events [25]; yet, a sensor or a place in the body for the sense of time remained enigmatic.

Moving in a physiological direction, Hippocrates has been credited for observing predictable, daily fluctuations in body temperature and illness severity [26]. However, not until centuries later, a closer look into the daily (circadian) rhythms of plants and animals was undertaken. Experiments by Jean-Jacques d’Ortous de Mairan in 1729, quoted in [27], showed that plants were capable of sticking to their normal rhythms despite being kept in continuous darkness, but his experimental design did not allow him to justly conclude that these patterns were produced internally since the lack of light alone does not rule out other possible external factors such as the Earth’s rotation or temperature.

Refinetti [26] reports that Condolle, in the early 1830s, alluded to the fact that the rhythmic force of the plants he used in his experiments must be internal due to the fact that their cycle was less than 24 hours – the reason being, that if the rhythmic forces relied solely on external/geophysical elements, the “clock” of the plants would be in synchrony with a single rotation of the Earth, i.e., equal to 24 hours [28]. By the 20^th^ century, experiments on activity patterns of animals [29-31] showed that animals like plants had a sense of time or “chronosense” [32] that operated in cycles quite close to, but not quite equal to, 24 hours [33].

It has since been known that the light-dark cycle of the Earth *entrains* the circadian rhythms of the organisms studied, whereby if the internal clocks of the species being investigated were allowed to run free, the pattern of behaviours would shift accordingly, relative to the solar-day [28]. The parameters, which entrain circadian rhythms are known as *Zeitgebers*[34,35] and can be biological or social (e.g. work schedules, appointments, deadlines, meal times etc.).

While circadian rhythms were being investigated, other researchers were forging insights into the human eye as the principle sensor of light. In the late 17^th^ century, the father of microbiology, Leeuwenhoek, was the first to observe the components we know today as rods and cones, but their photoreceptive properties were not discovered until the mid-19^th^ century [36]. Then, in the 1990s and early 2000s, researchers accumulated more and more evidence to support that the retina *in addition to the rods and cones* contains non-image forming photoreceptive cells with their own photopigments. The purpose of these new (or rather, “newly detected”, for they could be evolutionarily older than rods and cones [37]) photoreceptors has been an acclaimed research topic since their discovery. It has been shown that the photopigment melanopsin, [37-40] plays a significant role in the mammalian entrainment of circadian rhythms, independent of rods and cones [41-43] by providing signals to the suprachiasmatic nuclei via the retinohypothalamic tract [44]. It seems that apart from the known rods and cones there are other as yet unrecognized photosensitive structures, subsets of specific ganglion or bipolar cells, or overlooked mechanisms involved in the photoentrainment [41,44,45]. The inescapable conclusion is that the eye works as a dual sense organ, transmitting images to the brain, known traditionally as the sense of sight, but also monitoring time and being responsible for the sense of time of an individual.

To summarize, the non-scientist’s CS *that* “humans have a sense of time” and *that* “light is a strong determinant of one’s biological rhythms” has been proven right by modern science. If scientists now follow up causal relationships that involve the human sense of time and the circadian organization of our physiology, endocrinology, metabolism and behaviour, promising insights can be gained, for instance, into how some blind individuals, while being incapable to use light for sight, nevertheless exhibit the common spring/early summer suicide peak [Meyer-Rochow et al, in preparation] or benefit from “chronobiologically active radiation” [46].

(ii) Research into causes of seasonal affective disorders at extreme latitudes

With regard to humans, it has probably been CS since the dawn of time that light and its seasonal variations can affect how people feel and behave. Yet, surprisingly, behavioral and psychological changes in relation to the time of year – colloquially known as “winter depression” or “winter blues” and professionally referred to as seasonal affective disorder (SAD) – were not studied in a more systematic fashion until Rosenthal and colleagues [47] had formally described SAD in the 1980s. Triggers and mechanisms of SAD are not exactly known as it is difficult to pin-point specific causes of this newly established disorder. The fact that it has not been feasible, so far, to isolate any one of the variable components individually poses a key problem and challenge for research.

In 1986, Potkin et al. [48] hypothesized, based on the assumption that the development of SAD is distributed homogeneously throughout the study population and SAD is primarily caused by fluctuations in the period of daylight, that the prevalence of SAD increases with latitude. Using data collected from volunteers living throughout the United States, Potkin et al. [48] concluded “these data demonstrate the correlations among self-reported symptoms of seasonal affective disorder and latitude, December sunshine, cloudiness, and temperature”. With this observation, Potkin’s working group was credited for developing the SAD “latitude hypothesis” [49], which has since been examined through various methods.

Two studies conducted within the last decade [49,50] specifically investigated the relationship between latitude and SAD prevalence. Levitt and Boyle [48] stratified the province of Ontario, Canada (41.5°N to 49.5°) into parallel segments at increments of 1° latitude. Based on the eight resulting sectors, data on the prevalence of SAD were gathered through telephone interviews (n = 1,605), but no significant increases in the prevalence among residents living at higher latitudes were noticed. The findings of that study suggested that latitude had no apparent impact on the pattern of SAD’s occurrence. Brancaleoni et al. [49] also did not find a predominant correlation between latitude and the prevalence of SAD based on results of a survey distributed to students living in either Tromsø, Norway (69°N) or Ferrara, Italy (44°N). A literature review conducted in 1999 yielded similar results [51] and suicide data of victims from countries with distinct seasons also do not peak in the darkest season of the year, but rather in spring or early summer [52-54] despite a well established link between depression and suicide [55,56].

Yet the lack of a suggestive positive correlation between latitude and SAD in several of the studies conducted to date, does not mean that the latitude hypothesis should be dismissed altogether. First, confounding factors such as genetics [57,58], social organization, and economic well-being [59] may not have been given sufficient consideration in previous studies. For example, Brancaleoni et al. [49] surveyed students, but student populations typically consist of younger probands, who follow seasonal schedules that are, for the most part, out of their control within the scope of academic semesters, such as lectures and exams taking place on/at particular dates/times, all having the potential to affect the students’ seasonal body responses.

Second, aside from population life-style differences, the spectrum of latitudes that has been investigated in past studies may not capture the differential prevalence of SAD at higher latitudes. Going back to one of our examples, Levitt and Boyle [50] only looked at a range of 8° latitude. Perhaps the population they interviewed living at the southernmost latitudes already had a comparatively high prevalence rate of SAD and therefore no significant difference was detected when compared to populations living 8° further north. Third, as the results from a recent study conducted in Greenland [60] (64°N-77°N) may indicate, there could be a specific latitudinal threshold which is correlated with a significant increase in the prevalence of SAD. Kegel et al. [60] report an SAD frequency of 6.9% for the municipality of Nuuk (64°N), which has at least some daylight every day of the year, compared with a frequency of 11.5% in Uummannaq (70°N), which has eight weeks of polar nights. Although higher exposures to winter darkness may be associated with higher occurrences of SAD, such observations are not a *conditio sine qua non* as such requirement would, for instance, ignore the possibility of threshold phenomena.

Very recent experimental research in mice may help to establish the biological plausibility of latitude as a co-determinate of SAD from a developmental angle. Ciarleglio et al. [61] found evidence that the perinatal photoperiod may imprint the circadian clock in such a way that the alterations are still present in later stages of life. As a result of developmental gene–environment interactions, the development of the subject’s circadian clock to the seasonal photoperiods following birth appeared to determine circadian pacemaker responses to photoperiodic stimuli for the observed, albeit limited, later periods of life. There were positive effects (physiologically responsive and stable timing relationships to varying light/dark transitions that characterize seasonal changes) in subjects born under lighting conditions similar to summer periods, but rather negative ones (pathophysiologically unresponsive and unstable timing relationship to varying light/dark transitions that characterize seasonal changes) in those born under lighting conditions similar to winter periods. The authors found the behaviours in short day-matured mice to be in line with human SAD sufferers.

At extreme latitudes, the periods with winter lighting conditions are longer and more severe and, therefore – under the scenario suggested by Ciarleglio et al. [61] – can affect the development of more newborn humans than at lower latitudes. In other words, at extreme latitudes relatively more babies should be born during the long light-deficient winter season than, for instance, at the equator. If the reasoning provided by the researchers is valid, those born in the extreme northern or southern regions of the world shortly before or during the winter months could be expected to be at an increased risk of developing neurobehavioral disorders, such as SAD, later in life.

To summarize, the non-scientist’s CS *that* light can improve an individual’s mood and *that* seasonal deficits of light or extended winter darkness may lead to impaired mood and depression has found some, albeit ambiguous, scientific support via science in the past 25 years. While light therapy may be able to alleviate some of the symptoms of SAD, it is far from established that the less daylight one is exposed to (i.e., living at higher latitudes), the more likely one is to suffer from SAD. More such research will be needed to elucidate the precise nature of some probable, but presumably complex, causal links between exposure to ambient light and human health [60], in particular with regard to, but not confined to, mood disorders such as SAD. To date, no epidemiological study has investigated the promising lead that geography may co-determine the distribution of light-associated facets of health in human populations [46,62,63].

(iii) Research into the restorative and cancer protective functions of sleep

Hardly anyone would disagree with the non-scientist’s CS that sleep has abundant beneficial effects upon one’s health, and that sleep is indeed an essential factor in a person’s well-being.

Afternoon naps, so-called siestas (from *sexta hora* = the 6th hour) are found in cultures all over the world [64] and its most organized form in agricultural, settled societies rather than nomadic, hunter-gatherer cultures [65]. CS dictates that resting after meals is advantageous (hence “*plenus venter non studet libenter*”: freely translated from Latin “a fat belly, a lean brain”) and the well-documented afternoon accidents on roads as well as household peaks from many parts of the world underscore this notion [66,67]. Other negative impacts of sleep deprivation (e.g. impaired rationality, moodiness, physical weakness, and suppressed immunity) are recognized the world over.

More subtly, but equally valid, are the detrimental effects from inappropriate timing and conditions of sleep (e.g., sleeping outside the biological night time window and illumination: [68]). While there are several aspects, which have been identified links between poor sleep habits to illness, here we will focus on four interrelated facets of sleep that may have bearings on the risk of developing cancer. These sleep-related facets are DNA repair, inflammatory processes, melatonin titre, and so-called chronodisruption.

As early as 1960, one of the founders of chronobiology, Colin Pittendrigh implied that relevant disturbances of the timing of biological rhythms can lead to adverse health effects (“circadian rhythms are inherent in and pervade the living system to an extent that they are fundamental features of its organization; and to an extent that if deranged they impair it” [69]). A rather reductionist, but nevertheless interesting, detail of sleep’s possible role in protecting against cancer may have been elucidated in 2009. According to Greaves [70], mutations are necessary conditions for cancer. Some protection against critical accumulation and effects of such mutations is offered by DNA repair. Kang et al. [71] demonstrated that in experimental mice nucleotide excision repair follows a circadian rhythm. If we were to extrapolate these findings in nocturnal rodents to humans [72], it would not be unreasonable to expect that specific DNA repair is highest during our biological night, i.e., in the night/early morning hours or in the time window during which we regularly sleep. With this premise, it seems fair to suggest that appropriate sleep may allow or facilitate and that a disrupted and truncated sleep may disallow or impair protection against cancer development by preventing cells from repairing frequently occurring DNA damage.

Another perspective for understanding whether sleep could play a role in protecting against cancer may be provided by Simpson and Dinges [73] who report that there is a link between sleep loss and inflammatory processes. In fact, as described by Coussens and Werb [74], inflammation can directly and indirectly be associated with cancer.

It is known that the hormone melatonin is secreted primarily through the pineal gland during nighttime hours in the absence of light. In the presence of light, melatonin secretion is significantly reduced or blocked completely. It follows that melatonin is primarily produced and secreted during our times of sleep. Remarkably, melatonin can have effects on all six “hallmarks of cancer” [75]. In 2000 these two authors suggested that cancers are complex tissues determined by cascades of disrupted regulatory circuits, so that “a succession of genetic changes, each conferring one or another type of growth advantage, leads to the progressive conversion of normal human cells into cancer cells” [76]. It was claimed anticancer defence mechanisms fail and cells acquire some or all of the following six capabilities: (1) self-sufficiency in growth signals, (2) insensitivity to growth-inhibitory signals, (3) evasion of programmed cell death (apoptosis), (4) limitless replicative potential, (5) sustained angiogenesis (6) and tissue invasion and metastasis. Intriguingly, there is experimental evidence that melatonin, being primarily produced while we sleep, may protect against cancer by having favorable effects on all six hallmarks of cancer, namely with respect to proliferation processes [76,77], apoptosis [78-80], angiogenesis [81] and invasive and metastatic cell properties [76,82].

Today, it appears biologically plausible that chronodisruption, described as a significant disruption of the coordination and thus order of biological rhythms and effects, could contribute to the development of cancers [83,84]. Since melatonin is credited with anti-cancer properties, inhibiting tumorigenesis and hindering tumour growth [46,85-87], it has been hypothesized that sleep duration is negatively correlated with the occurrence of hormonal cancer cases, such as breast cancer [62,88,89]. In this vein, Reiter et al. [86] state “a number of studies have reported an inverse correlation between the frequency of cancer in the population and duration of sleep; thus in most studies an elevated cancer risk was associated with shorter sleep intervals”. This observation has been extended to individuals who work in shifts, compromising the traditional and physiological periods of sleep, and thereby increasing the risk of developing certain types of cancers [90].

The non-scientist’s CS *that* sleep is a cure for many ills is therefore finding increasing scientific support. However, precisely *how* sleep renders these beneficial health effects, despite extensive research from a number of different biomedical angles, remains an open question. Based on light- and shift-work data gathered from both epidemiological and experimental settings the act of sleep alone should not be expected to be the all-conditional healer, or *the* preventive means to avoid cancer, because factors surrounding sleep could be relevant as well. It may be crucial to respect, rather than abuse, restorative patterns of physiology that have evolved in the human biological system long before the industrial revolution and the advent of artificial lighting have led us to radically alter the length and time of day and our activities. Insights into the age-old wisdom that sleep is a necessity for good health have shown that not only sleep duration but also the conditions under which we sleep can affect our health.

To summarize, it can be expected that in the future both epidemiologic and experimental research have the potential to grow appreciably in scope and scale by focusing on areas that CS has identified for some time as key ingredients to physiological restoration and health.

(iv) Research into links between social networks and health and disease

The term “social networks” and analyses of their benefits for individuals may be little older than a century. However, the concept *that* good friendships and being embedded in social groups contribute positively to an individual’s well-being and health has been CS for millennia and been reiterated very recently in an article on happiness (Time Magazine, July 2013, double issue). Conversely, lack of social networks and lack of their support are generally expected to be linked with a host of negative effects on one’s health. In this vein, pursuing scientific answers to the question as to whether and, more importantly, *how* social networks may influence health endpoints constitutes an important example of where the non-scientist’s CS may be considered to be of guiding influence.

One root of the question goes back to Leonard Syme, one of the pioneers of social epidemiology in the past 50 years and who was interested in the power of social networks in the early 1950s [91]. Pivotal work in those years and the 1970s illustrated that group behaviour can determine individual “health outcomes”, including suicide [91-93]. According to Syme [91], important scientific insights into the links between “social networks” and “social connections” with health and disease actually “resonate with common experience”. However, while Syme wrote that “the importance of social networks has become a recognized international fact” [91], the questions as to 'important for what’, 'how’ and 'to what extent’ appear to be still unanswered as evinced by the controversies around recent empirical studies.

One of the two protagonists of research in the 2000s into the question of 'how social networks contribute to health in populations’ [94,95] was actually challenged by his very wife (acting, if you will, as a CS advocate) that he should not dare waste precious taxpayers money by investigating a question, which had such an obvious answer: “of course friends influence one another” [96]. But despite pivotal work in the second half of the last century [91] and powerful databases, the ultimate verdict on this question is still out. While science may be looking into what the non-scientist’s CS “knew all along”, getting to the heart of the conceived causal relationships may prove very tricky and require extensive work, first recognized and explained by Fleck [97].

To exemplify, two of several effects claimed to be related to social networks, namely the distribution of obesity and smoking, have been examined by Christakis and Fowler by using data collected within the Framingham Heart Study, launched in 1948. Analyses of data gathered on the dense social network reconstructed by using information repeatedly collected from more than 12,000 individuals over 32 years up to the year 2003, evinced – according to the two authors – that person-to-person spread may have critical roles for the increase of obesity [94] on the one hand, and for the decrease of smoking [95] on the other. And yet, what haunts epidemiologists whenever they try to infer causality from observations [98] is critical here as well: do the Framingham cohort study results show mere associations or do they reflect causality? In other words, does the mere identification of a health pattern among socially connected people imply, let alone prove, that one person causes other persons to do something and thus causes health outcomes or not? In addition, even if there were not just associations but cause-and-effect links between social networks and health and disease, what trends do they actually follow? In principle, social networks could be the result rather than the cause of health and disease. The vivid exchange of correspondence in the *New England Journal of Medicine*[94,99-101] can be seen as the prelude to continued discussions and further research.

In any case, the non-scientist’s CS that there *are* links between social networks and health and disease in mankind appears obvious and persuasive. And yet, scientists need to continue to carry out rigorous studies to ultimately determine whether, and more importantly, *how* social networks, health and disease are related.

To summarize, large populations and scientists alike would subscribe to the notion that the traditional sense of social networks are a promising CS lead into possible key determinants of health and disease. Going back to Syme [91], a good example of how tantalizing this notion is, and how one takes its validity for granted is his confession:

“I had no interest in the topic of mental illness because I took it for granted that social factors would somehow be related to mental illness. Looking back, I can see what a naïve view this was, but that was my uninformed position at that time. Instead, I wanted to know if social factors were related to diseases that were not so obviously connected to the social world, diseases such as heart disease, cancer and arthritis.”

Syme had taken the CS of social factors influencing mental illness for what it appears to be, namely a relationship that *does* exist - leaving the exact mechanisms which propel the relationship between mental health and social networks uncovered.

## Discussion

(a) Common sense may not always make sense

We have to expect that CS can and will sometimes be based on beliefs lacking corroboration by science. This, however, should not prevent us from seeking answers. CS dictated that the Earth was flat, but this was shown to be wrong. CS also advised someone in a thunderstorm with this piece of folk wisdom: “Beach trees you look for, oak tress you ignore”. It was thought that oak trees possess deeper roots and therefore attract lightning, but it is now known that lightning strikes both tree types equally. There are many more such examples, in which CS did not make sense when tested. What we propose is that the links suggested by CS be examined by scientists for their causality. Provided the latter can be confirmed, it may then lead us to elucidate with additional rigorous research how links in the chain of causality may be broken in order to find novel approaches to improve public health and health care.

(b) Common sense may not be testable at all

In terms of mistaken CS convictions or beliefs, it might – and presumably will – be argued by some readers that CS lives on for generations, because what it suggests is not scientifically testable, but “culturally based reasoning” [102]. We have our doubts about this and believe CS is usually not taken seriously enough and consequently it is not tested. In many instances to show *that* the causal link is or is not there should take relatively little time. Moreover, hypotheses which are properly formulated are testable, according to Popper.

(c) Common sense is often greeted skeptically by scientists

Referring to the lack of scientific proof, scientists usually greet CS with a great deal of skepticism, even hostility. At best they view CS as an assumption that might be worth consideration. Scientists are trained that assumptions can be misleading and therefore can take them into dangerous terrain. In fact, Einstein is often quoted to have said *“Common sense is the collection of prejudices acquired by age eighteen“*[103]. However, he also is quoted as saying *“Common sense invents and constructs no less in its own field than science does in its domain.”*[11]. This demonstrates the ambivalent stance the scientist finds him/herself in when confronted with CS.

Yet, as we tried to explain in this paper, CS should be valued as a potential source for innovative research. Those scientists open-minded enough and willing to follow up and test CS assertions may either get the satisfaction of having disproven a particular CS assertion (which could also be an important finding) or they end up reaping the benefit of some long-held but unproven belief.

(d) Which common sense themes might be worthwhile to be pursued?

In the context of our focus, CS suggestions would have to refer to the natural sciences, to medicine and sociology, but clearly not to political, religious or belief systems that involve issues likes ethics or morality. Rather, scientists, for instance in the ethnobiological and medical field, may ask themselves “if this or that CS can be shown to be true, would understanding how A leads to B and how to manipulate the causal chain between A and B make a difference to our understanding of local attitudes?”

Pursuing CS leads may sometimes seem ridiculous, perhaps inappropriate or even dangerous as the very research into the possible validity of CS may erroneously suggest credibility where none is due. The latter could apply to political views, or religious beliefs and sentiments, which is why we exempted them (see above). However, it seems safe and warranted to test those suggested causal links and phenomena which, if truly causal, could make a difference in improving health or living conditions. Therefore, to simply ignore CS seems a recipe for failure or delay of progress and a disservice to innovative science. We quote: “If speculations are wrong they attract no following and disappear. If they are right they can be the beginning of new fields of knowledge” [104]).

(e) Teleological considerations suggest that pursuing common sense “makes sense”

Two critical considerations are in order here: first, irrespective of being “right” or “wrong”, as long as aiming to falsify CS hypotheses leads to rigorous tests we all, scientists and non-scientists, shall benefit. A historical example may illustrate this important consideration empirically. When there was some intellectual controversy in the 1930s as to whether information was transmitted from cell to cell via electrical or chemical ways, the 1963 Nobel laureate Sir John Eccles chose the wrong side. However, his rigorous opposition to the theory of chemical transmission forced scientists to collect more and more convincing data.

Second, who is able to determine beforehand which CS conviction or belief will be proven wrong, in detail or in context? It seems actually appropriate to discuss improbable and perhaps even faintly ridiculous CS and have it tested, and subsequently vindicated or destroyed. Once again, as Horrobin, quoting Popper, writes “If a hypothesis which most people think is probably true does turn out to be true (or rather is not falsified by crucial and valid experimental tests) then little progress has been made. If a hypothesis which most think is improbable turns out to be true, then a scientific revolution occurs and progress is dramatic” [21].

A case in point is the “tummy bug”: a CS notion that some microorganism resided in the stomach and gave you some stomach trouble. Rejected for years on end and put down to “stress”, it was the research by Barry Marshall and Robyn Warren that finally proved that stomach ulcers were indeed caused by an organism, namely *Helicobacter pylori*[105]. The two researchers were awarded the Nobel Prize in 2005 for their work.

Cold causes colds is another such example. Dismissed by generations of physicians as nonsense, because only microorganisms and not the cold weather or the sensation of feeling cold can cause the common winter respiratory tract infections with sore throats and running noses, it has recently been shown that cold weather stress leads to vasoconstrictions in the respiratory tract mucosa and suppression of immune responses. The consequence is an increased susceptibility to infections when feeling cold [106].

(f) Revisiting the common sense of scientists

Advocating that more attention be awarded to non-scientists’ CS is not to say that the CS of a scientist is unimportant. However, the CS of scientists may be misleading for reasons of vested interests, but also because to scientists, “common sense” has a non-scientific, and thus negative, connotation. Yet, in our view CS, as demonstrated through the examples above, can be a springboard for innovative science. That is not to say that all aspects of CS will prove to be true or will stand the test of time as CS must not be regarded as something ever-lasting. CS changes over time as new information becomes available and is communicated to and distributed throughout the public. What once may have been considered “common sense” or “common knowledge” may be considered non-sense or even ignorance today.

(g) How can common sense research contribute to the scientific fields of ethnobiology and ethnomedicine?

Ethnobiologists and investigators of medicinal practices and beliefs traditionally observe, listen, record, and describe. The views expressed to them by their informants may not be based on sound scientific knowledge, but grounded in what might be called 'folk wisdom’. When one of the authors (VBM-R), married to a Brahmin Hindu, wanted to pick a lemon off a tree in the garden early at night, his wife told him that he could not do that. Inquiring for the reason and pointing out it was not fully dark yet, he was told that it was common sense that plants (being living organisms albeit of lower consciousness) also required some rest and therefore should not be disturbed in the dark.

One could file this experience under religious beliefs and explain it metaphysically, but one could also launch an inquiry into the question of consciousness in plants and its scientific basis or investigate whether this application of common sense had its origin in the fact that human vision at night is poor and that injuries or attacks by dangerous animals are more likely at that time of day or even whether disturbances of plants at night stunt the plants’ growth or affect them in some way.

What this example and others given in the introduction to this review article show is that ethnobiological and ethnomedical researchers should not only see the need to record common sense statements in one community or ethnic entity, but ought to compare similar statements from different regions, paying attention to the social and physical environment that common sense beliefs had evolved in. Follow-ups, if necessary in collaboration with experts of relevant other disciplines, should then be aimed to scrutinize the validity of the common sense statements and to shed light on the question as to why common sense statements (just like taboos [25]) sometimes are shared between neighbouring communities, but often also differ between one community or geographic region and another [107]. In our view the advocated greater attention to common sense by ethnobiological and ethnomedical researchers in collaboration with other scientists would undoubtedly lead to a deeper understanding of certain specific human behaviours, beliefs, and practices that one is frequently confronted with (and sometimes puzzled of) in one’s investigations.

## Conclusions

Accepting that CS of non-scientists can be beneficial to the scientific community, the following conclusions are warranted: Non-scientists should not only be viewed as sources of information, but as full-fledged collaborators in the research on CS and its potential for scientific exploration. CS is built on observations and powerful tests of hypotheses made by large populations. Put differently, scientists should pay attention to non-scientists’ CS observations, explanations and predictions, because usually they have survived a plethora of tests and have undergone numerous variations and refinements. This is particularly true with regard to folk-medicinal practices and views on health-promoting behaviours.

While non-experts certainly differ from experts in their background and training to observe and test, this should not necessarily be viewed as a negative or knock-out criterion [15]. Importantly, vested interests do not necessarily compromise objectivity in non-scientists. Moreover, the number of non-expert “observers” and “experimenters” exceeds the number of scientists by many orders of magnitude. It is here, that a phenomenon such as “group intelligence” may add additional weight to CS ideas, convictions and rationale. Intriguingly, group intelligence in itself is a good non-scientist-CS candidate for targeted research [108,109]: some evidence appears to point to the fact that group thinking has much value, but science fails to understand whether and, if so, how this really works.

As one practical consequence of the predictive power of the non-scientist’s CS we may want to use CS convictions of causal relationships in everyday life regardless of whether scientific explorations into its nature have already been made or not [110,111]. To exemplify, as sleep hygiene is a CS belief, one should arrange for sufficient and appropriate sleep, irrespective of what the precise health benefits and mechanisms are. Similarly, as cross-cultural investigations have shown [112] friendships and social networks should be nurtured by all means, including mobile phones [113], irrespective of the fact that we lack details as to how social networks can be critical for health and disease. But if research were to substantiate *that* causal links predicted by the non-scientist’s CS are valid, then we should *a forteriori* use CS convictions for preventive purposes if there are means to do so.

With regard to innovative science, researchers should watch out for the non-scientists’ CS suggesting *that* A is associated with or causes B. However improbable, on the one hand, or self-evident, on the other, one or more CS convictions might appear in a researcher’s view and elucidation of *how* A relates to B could be the next big thing in science. In order to seek clues of what is important for the public and what should be studied with some priority, it is time to try to identify causal links that are worthy of rigorous investigations with an emphasis on the non-scientists’ CS. Surely, this cannot be worse than being exclusively led by scientists, whose vision may be obscured by vested interests. Indeed, as evinced by the examples presented in this paper, the non-scientist’s CS may do much better. A very recent study [114], in which it was argued that partially due to “common sense” Norwegian fishermen did not see a need to formalize regulations to improve their safety, underscores this point. Following up CS leads, backed by rigorous research, may ultimately contribute to a better understanding and control of the natural and man-made world around us.

## Competing interests

The authors declare that they have no competing interests.

## Authors’ contributions

TC Erren started this work as a visiting scholar at the UC Berkeley in 2010; M Koch assisted with the literature survey in Germany and together with TCE prepared the first draft; VBM-R added information and completed the manuscript. All authors read and approved the final manuscript.
